# Immediate Return to Ambulation and Improved Functional Capacity for Rehabilitation in Complex Regional Pain Syndrome following Early Implantation of a Spinal Cord Stimulation System

**DOI:** 10.1155/2014/784021

**Published:** 2014-11-24

**Authors:** Brandon Jesse Goff, Jeremy Wingseng Naber, John Patrick McCallin, Edward Michael Lopez, Kevin Brant Guthmiller, Karl Alan Lautenschlager, Tristan Toll Lai, Dean Harry Hommer, Gonzalez Raul Marin

**Affiliations:** ^1^San Antonio Military Medical Center, San Antonio, TX 78234, USA; ^2^Philadelphia College of Osteopathic Medicine, Philadelphia, PA 19128, USA

## Abstract

Complex regional pain syndrome (CRPS) is a neuropathic pain condition that is characterized by vasomotor, sensory, sudomotor, and motor symptoms. Spinal cord stimulation (SCS) has been successfully utilized for the treatment of pain refractory to conventional therapies. We present a case of a previously highly functioning 54-year-old female who developed a rarely reported case of idiopathic CRPS of the right ankle which spontaneously occurred four months after an uncomplicated anterior cervical disc fusion. This condition resulted in severe pain and functional impairment that was unresponsive to pharmacological management. The patient's rehabilitation was severely stymied by her excruciating pain. However, with the initiation of spinal cord stimulation, her pain was adequately controlled allowing for progression to full unassisted ambulation, advancing functional capacity, and improving quality of life. This case report supports the concept that rapid progression to neuromodulation, rather than delays that occur due to attempts at serial sympathetic blocks, may better control symptoms leading allowing for a more meaningful recovery.

## 1. Introduction

Complex regional pain syndrome (CRPS) is a neuropathic pain condition characterized by sensory, vasomotor, sudomotor, and motor/trophic signs and symptoms [1]. CRPS type 2 formerly known as causalgia is preceded by a major nerve injury. CRPS type 1 formerly known as reflex sympathetic dystrophy presents with similar symptoms as type 2 with the absence of a major nerve injury. It usually is preceded by some trauma to the limb [2]. Although the pathophysiology of this condition is not well understood, it is often preceded by a trauma or an operation on an extremity [3]. The incidence of CRPS has been reported as 5.46 cases per 100,000 persons, with increased frequency in women [4, 5]. CRPS resolves within the first year after diagnosis in 70–85% of patients, but in the remaining 15–20% becomes refractory to treatment [4]. Currently there are no criteria for identifying those patients who will progress to the chronic form of CRPS, although sensory disturbance and cold skin temperature are associated with poor prognosis [6]. In patients who have CRPS chronically, the condition can be considerably debilitating. Extreme pain, low functionality, and permanent disability significantly affect not only the quality of life for those with CRPS, but also the lives of the families [7, 8].

Overall, treatment of CRPS has produced mixed results, with no scientifically validated treatment. Some of the treatment modalities that have been used to treat CRPS include physical therapy, occupational therapy, conventional pain medications, antidepressants, anticonvulsants, opioids, ketamine infusions, sympathetic blocks, transcutaneous electrical stimulation, and spinal cord stimulators [9, 10]. Spinal cord stimulators (SCS) are often tried only after other treatment modalities have failed to produce positive results. Used for the treatment of chronic pain since 1967, spinal cord stimulators have proven effectiveness in treatment of the burning pain associated with CRPS, but less evidence for improvement in limb functionality, and reduction in allodynia and hyperesthesia [11, 12]. When using a SCS, an electrode is percutaneously or surgically placed into the epidural space on the dorsal side of the spinal cord corresponding to the level of the nerve roots supplying innervations to the area with pain. The current supplied by the electrodes induces paresthesias and suppresses pain sensation in the affected limbs, although production of paresthesias over the affected body part is not required for pain relief and functional improvement [1, 13].

Implanting a SCS is often considered both an expensive and an invasive treatment, and satisfactory lead placement is necessary for successful treatment. On the other hand, despite the apparent upfront cost, if the treatment is appropriate and is shown to have good outcomes, overall costs, morbidity, and chronic decreased functionality would be significantly reduced with fewer ineffective treatments and tests. Studies in both the US and in the United Kingdom comparing the use of SCS with conventional management alone determined that, in selected patients with CRPS, SCS is cost-effective [2, 14]. We present a case of a woman with CRPS type 1 refractory to conservative efforts, who was treated with early spinal cord stimulation, foregoing the traditional, and recommended time line of interventions and found to have improved ambulation and functionality of the affected limb immediately and over the next six months.

## 2. Case Presentation

**Figure 1:**
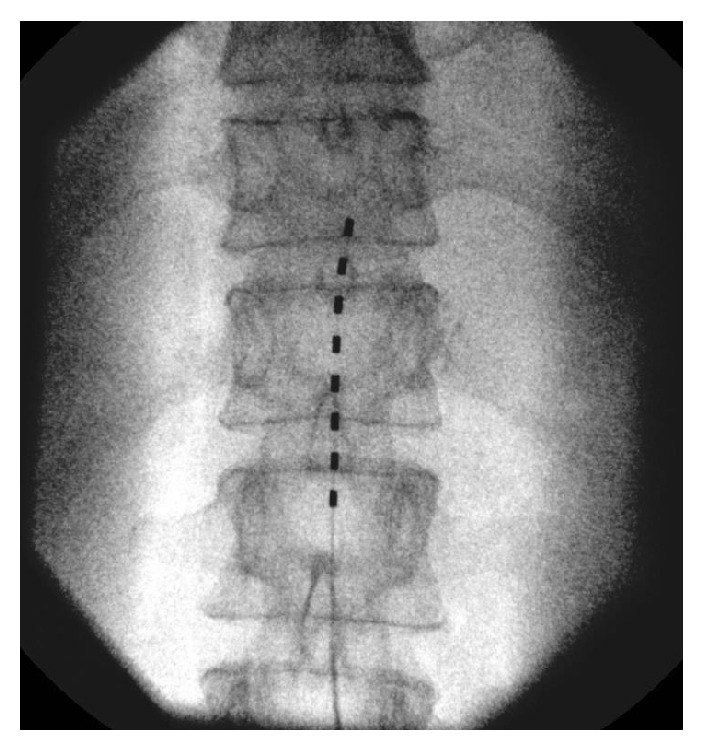
AP fluoroscopic view of SC trial placement.

**Figure 2:**
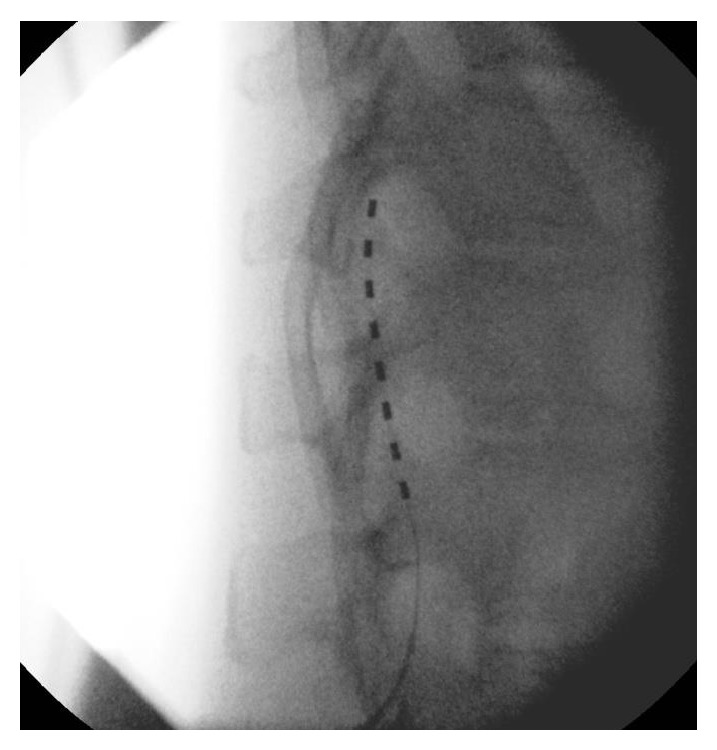
Lateral fluoroscopic view of SCS trial.

**Figure 3:**
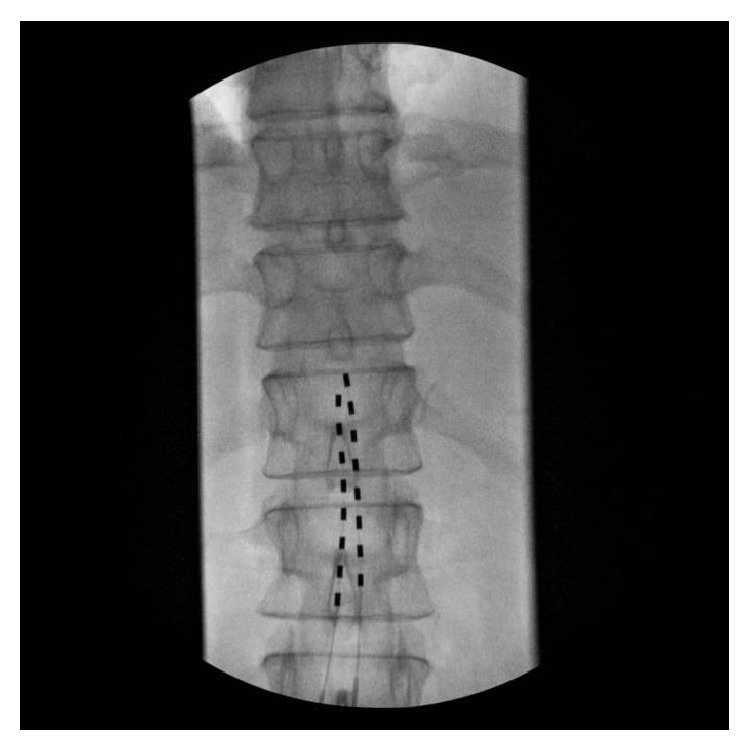
AP fluoroscopic view of SCS implantation.

**Figure 4:**
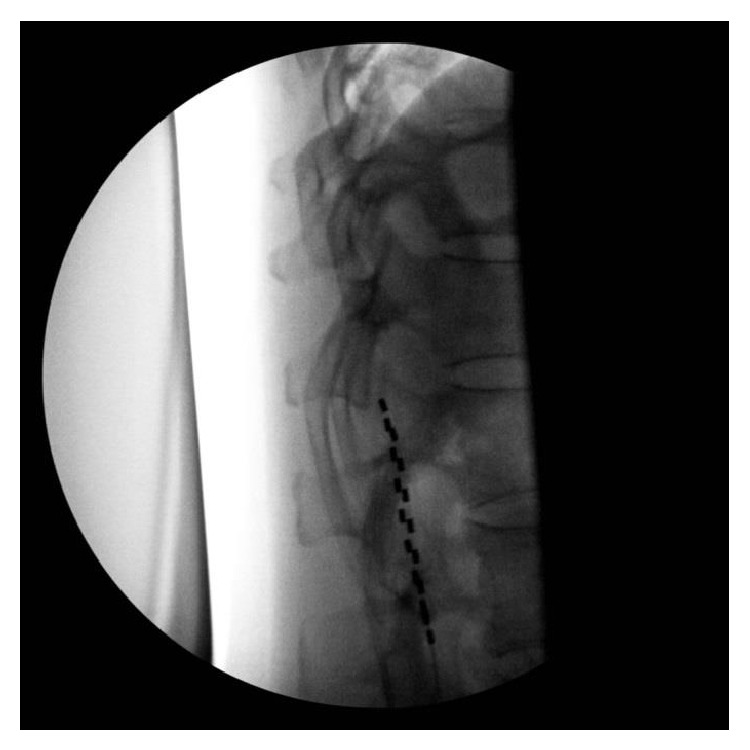
Lateral fluoroscopic view of SCS implantation.

The patient is a 54-year-old, outdoors-oriented woman who began to experience intermittent pain, paresthesias, and numbness of the right arm followed by the same symptoms in the bilateral lower extremities. Within 4–6 weeks she was only able to walk with assistance and then limited to a wheelchair. The patient had an MRI of her brain and spinal cord to rule out multiple sclerosis (MS). Her brain MRI revealed some white matter lesions that were not consistent with MS, but her C-spine MRI showed a significant C5-6 herniated nucleus pulposus and severe canal stenosis at C5-6, which flattened her spinal cord. This pathology was thought to be the cause of her symptoms. Subsequently, she underwent a C4–C7 anterior cervical discectomy and fusion surgery. The surgery appeared successful as she was ambulating independently within 1 week of surgery and hiking long distances at altitude within 6 weeks. At this point, the patient did not have any physical limitations or deficits. Four months after surgery, she began to experience unexplained pain, paresthesias, and numbness in her right foot/ankle. This quickly became an uncontrollable pain requiring axillary crutches and then a wheelchair. After three months of the foot/ankle pain, she was seen by Physical Medicine and Rehabilitation and was then enrolled in a comprehensive rehabilitation program for presumed CRPS. Even though she was treated with opioid and neuropathic pain medications (morphine sulfate, gabapentin, pregabalin, and amitriptyline), she was unable to participate fully in physical therapy due to pain. During month 4 of foot/ankle symptoms, the pain worsened and she was no longer able to bear weight on her right lower extremity and the foot/ankle began to exhibit classic signs and symptoms of CRPS including allodynia, edema, erythema, hyperhidrosis, decreased range of motion, and dystonia [15, 16]. Electrodiagnostic studies showed no evidence of large fiber neuropathy, distal tibial neuropathy, or right lower lumbosacral radiculopathy. However, testing revealed significant asymmetry in skin temperature, 29.0 Celsius on the left lower extremity and 26.3 Celsius on the right, along with asymmetrically prolonged onset latency difference of 484 msec on the right. She was expedited to the Department of Pain Management for sympathetic blocks, but it was decided to pursue early neuromodulation instead. Approximately 4-5 months after the onset of symptoms she underwent successful SCS trial. The trial was performed in the pain management clinic and consisted of the placement of one percutaneous lead with eight electrodes introduced via a 14 gauge modified Tuohy needle and entering the epidural space in a parasagital approach at L2-3 under fluoroscopy. Lead location in the posterior epidural space was confirmed with lateral fluoroscopy. The trial lead was advanced carefully to the T11-12 disc space under intermittent fluoroscopy (Figures 1 and 2). The patient reported incredible pain relief and that she was ambulating around her home for the first time in months. The paresthesias over her most painful areas allowed her to regain the majority of previously lost function and she began to take fewer medications. Because of the unqualified success of the trial, she went on to successful implantation in the operating room of two percutaneous leads with eight electrodes placed in the same area as the trial. For the implantation, two leads were used instead of one in order to maximize potential programming ability (Figures 3 and 4). She reported that the induced paresthesias allowed her to stand and ambulate around her home for the first time in months and to begin actively participating in intensive physical and occupational therapy. After placement of the SCS, the patient was seen for physical and occupational therapy sessions of one hour each, five times per week, for four weeks. Sessions were then decreased to one hour each, three times per week, for fourteen weeks. Physical therapy consisted of graduated weight-bearing, gait training, stepping and balance drills, recumbent cycling, standing elliptical, and lower extremity strengthening exercises. Occupational therapy consisted of desensitization, treatment with IFC (inferential current), and graded motor imagery. The patient was seen regularly by a Physical Medicine and Rehabilitation physician to monitor symptoms and adjust medication and functional therapy as needed. She was also seen regularly in the Department of Pain Management for follow-up of the SCS and an IFC unit was prescribed for home use as well. In addition, the patient was seen weekly by a behavioral health psychologist (also within the Department of Pain Management) for cognitive behavioral therapy and biofeedback. Finally, the patient participated in an 8-week course of mindfulness-based stress reduction. Ten months after the onset of symptoms she is no longer taking pain medication, is independent in all ADLs, and has returned to work.

## 3. Discussion

The treatment of CRPS requires prompt diagnosis and timely referral to avoid secondary morbidity related to disuse of the affected limb and to reduce the risk of developing a refractory, chronic form of the disorder [17]. Current recommendations for initial treatment of CRPS include integrated multidisciplinary management, pain control with oral medications such as anticonvulsants, tricyclic antidepressants, or opioids, physical and occupational therapy, and psychological support including cognitive behavioral therapy [12, 16–18]. More invasive interventions are generally reserved for refractory cases. Recently in the literature there has been some discussion about using spinal cord stimulation earlier in the treatment of CRPS [2] Poree et al. state that in some cases, SCS for the treatment of chronic pain of CRPS is “safe, appropriate, more cost effective with a relatively low time to fiscal neutrality, and as effective or even more effective when compared with chronic opioid maintenance or conservative management.”

This particular patient had early mobilization and treatment with opioids and anticonvulsants. After these measures failed, she was sent to the Department of Pain Management for consideration of a sympathetic nerve block. However, evidence-based reviews suggest that sympathetic nerve blocks are not effective in reducing CRPS pain or at best have only transient effects [19]. In addition, it was felt that time spent pursuing ineffective treatment could delay adequate mobilization and allow the disorder to progress to a chronic state. It was decided to proceed directly to a SCS trial. The pain control then provided by successful implantation of a SCS was sufficient for the patient to begin actively engaging in mobilization and desensitization therapies. While it is likely that the comprehensive treatments she received after SCS implant were key to restoration of function, adequate pain control was the initial priority and the catalyst which facilitated rehabilitation.

Although this is a single case report, further review of the literature and/or small clinical trials should be considered to see how effective early treatment with neuromodulation therapy can be for CRPS I patients. This endeavor would be worthwhile because the goal of treatment is to get patients back to normal functioning as quickly as possible.
